# Myo5B plays a significant role in the hyphal growth and virulence of the human pathogenic fungus Mucor lusitanicus

**DOI:** 10.1099/mic.0.001482

**Published:** 2024-07-29

**Authors:** Trung Anh Trieu, Lam Minh Duong, Phuong Anh Nguyen, Thuoc Van Doan, Hung Phuc Nguyen

**Affiliations:** 1 Faculty of Biology, Hanoi National University of Education, 136 Xuan Thuy, Cau Giay, Hanoi, Vietnam

**Keywords:** dimorphism, fungal infection, *Mucor lusitanicus*, mucormycosis, *myo5B*, myosin V

## Abstract

Mucormycosis is an emerging and deadly invasive fungal infection caused by fungi belonging to the Mucorales order. We investigated the myosin superfamily, which encompasses diverse actin-based motor proteins with various cellular functions. Specifically, the role of the Myo5B (ID 179665) protein from the myosin class V family in *Mucor lusitanicus* was explored by generating silencing phenotypes and null mutants corresponding to the *myo5B* gene. Silencing fungal transformants exhibited a markedly reduced growth rate and a nearly complete absence of sporulation compared to the wild-type strain. The *myo5BΔ* null mutant strain displayed atypical characteristics, including abnormally short septa and inflated hyphae. Notably, there were a majority of small yeast-like cells instead of filamentous hyphae in the mutant. These yeast-like cells cannot germinate normally, resulting in a loss of polarity. *In vivo* virulence assays conducted in the *Galleria mellonella* invertebrate model revealed that the *myo5BΔ* mutant strain was avirulent. These findings shed light on the crucial contributions of the Myo5B protein to the dimorphism and pathogenicity of *M. lusitanicus*. Therefore, the myosin V family is a potential target for future therapeutic interventions aimed at treating mucormycosis.

## Data Summary

The authors confirm that all supporting data, codes and protocols have been provided within the article or through supplementary data files.

## Introduction

Mucormycosis, formerly known as zygomycosis, is a potentially deadly invasive fungal infection caused by species of the Mucorales order. While it primarily affects immunosuppressed patients, immunocompetent individuals with severe injuries that compromise the skin or mucosal barriers can also be susceptible. The disease is characterized by rapid development, resulting in thrombosis, tissue infarction and subsequent necrosis due to the invasive destruction of blood vessels and vital organs [1,2].

Effective management of mucormycosis requires prompt diagnosis, identification and mitigation of predisposing factors; surgical debridement and timely administration of appropriate antifungal agents. However, due to the inherent difficulties in diagnosing and treating this condition, particularly in its early stages, controlling mucormycosis presents a substantial challenge [3]. The mortality rate associated with this infection is alarmingly high, ranging from 31 to 96 %, and is contingent on various factors, such as underlying risk factors, site of infection, type of pathogen and treatment administered [4,5]. Despite being considered a rare condition, the global incidence of mucormycosis is on the rise, with rates varying from 0.005 to 1.7 cases per million population. Notably, India has reported an incidence rate 80 times higher than the average (0.14 per 1000 individuals) [1]. The coronavirus disease 2019 (COVID-19) outbreak has resulted in a substantial increase in mucormycosis cases, especially in India, where over 47,000 cases were reported within a span of 3 months (May to July 2021) during the second wave of the pandemic [6]. Therefore, there is an urgent need to deepen our understanding of the pathogenesis and molecular mechanisms of mucormycosis and COVID-19-associated mucormycosis to effectively control this emerging infection.

Virulence factors play a crucial role in the invasion and damage caused by *Mucorales* fungi that cause mucormycosis. The key factors include the high-affinity iron uptake system, the dimorphism mechanism and azole resistance. Dimorphism is one of the main features of pathogenic fungi, particularly human fungi. The virulence of the yeast phase is lower than that of filamentous hyphae [7,8]. Recent advances in molecular and genetic tools have enabled the characterization of new genes, pathways and molecular mechanisms that control the pathogenic potential of *Mucorales* and their interactions with hosts [8,12]. The discovery and utilization of RNA interference (RNAi) in the dimorphic fungus, *Mucor lusitanicus*, a model for studying mucormycosis, have facilitated rapid screening and identification of virulence factors [13,16]. In a previous study, *mcplD* and *mcmyo5* genes, encoding phospholipase D and myosin V proteins, respectively, were identified as critical for the proliferation of the fungus and effective infection [17].

Myosins, a broad superfamily of actin-based motor proteins, play an important role in numerous cellular activities. The structure of myosin proteins comprises three domains: the N-terminal head, which includes the motor domain (an ATP-binding region that interacts with actin); the neck domain (which binds to light chains or calmodulin) and the C-terminal tail region (which anchors and positions the motor domain for interaction with actin) [18]. In filamentous fungi, there are four classes of myosins: myosin I, myosin II, myosin V and fungus-specific chitin synthase (Chs) with myosin motor domains. Myosin class V proteins, which are double-headed motors that facilitate vesicle transport, are vital for preserving cell polarity. Previous research has shown that class V myosin is essential for tip growth, cellular polarization and virulence in pathogenic fungi, such as *Candida albicans*, *Aspergillus nidulans*, *Ustilago maydis* and *Aspergillus fumigatus* [19,24].

In *M. lusitanicus*, loss of *mcmyo5* was previously shown to substantially reduce growth rate, sporulation and virulence [17]. This study was carried out to explore the function of the Myo5B protein, a member of myosin V, in the morphogenesis and pathogenesis of the pathogenic fungus *M. lusitanicus*. The findings could contribute to our understanding of the function of this protein family and the development of future effective therapies to treat mucormycosis.

## Methods

### Strains, growth and transformation conditions

The fungal mutants were generated using MU402 (*leuA*^-^ and *pyrG*^-^) as a recipient strain for the knockout experiment. Leucine auxotroph R7B (*leuA^-^*), a derivative of *M. lusitanicus* CBS277.49, served as the wild-type strain [25]. Fungal cultures were cultivated at 26 °C in minimum yeast nitrogenous base (YNB) medium, complete yeast–peptone–glucose (YPG) medium or selective minimal media with casamino acids (MMC). The pH was adjusted to 4.5 and 3.2 for mycelial and colonial growth, respectively. Uridine (200 µg ml^−1^) was added when necessary. In the transformation experiments using protoplasts, the medium was supplemented with 0.5 M sorbitol to prevent cytolysis caused by the osmotic gradient [25].

Cloning experiments involving *Escherichia coli* strain DH5α were conducted at 37 °C in Luria Broth (LB) medium (pH 7–7.4) using the heat shock method [26]. Ampicillin (100 µg ml^−1^) was used as a medium for transformant selection.

### Plasmids

To construct RNAi plasmids with the target candidate genes, plasmid pMAT1812 was used as a cloning vector [26]. A 2 kb fragment close to the 5’ end of the *myo5B* gene was PCR amplified using oligo pair Fsl-1 and Rsl-1 (Table S1, available in the online Supplementary Material). This fragment was cloned into pMAT1812 to generate the RNAi plasmid pAT31, which interfered with the expression of the *myo5B* gene (Table S2).

Plasmid pAT47 was constructed to generate a disruption cassette for the target gene deletion. The disruption cassette containing the selective marker, *pyrG*, flanking the upstream and downstream regions of the *myo5B* gene (~ 1 kb each) was cloned into pJET1.2/blunt (Thermo Fisher Scientific, Waltham, MA, USA). The sequences of the DNA oligonucleotides used in this study are listed in Table S1. Plasmids were generated as previously described [26].

### DNA manipulation and analysis

The *myo5B* (ID 179665) sequences and characteristics were obtained from the genomic database of *M. lusitanicus* CBS277.49, accessible through MycoCosm (the fungal genomics resource) at the Joint Genome Institute, US Department of Energy [27]. Genomic DNA from *M. lusitanicus* was extracted from the mycelia using a Fungi/Yeast Genomic DNA Isolation Kit (NORGEN Biotek, Canada). Bacterial plasmid extractions were carried out using the GenElute Plasmid Miniprep Kit (Sigma-Aldrich, Germany), following the manufacturer’s instructions.

PCR reactions utilized Phusion High-Fidelity DNA Polymerase (2 U/µL) from Thermo Fisher Scientific, with PCR mixture preparations and thermocycling programme settings in accordance with the manufacturer’s guidelines. Purification of DNA samples was achieved through direct purification or agarose 1 % electrophoresis, employing the GenElute PCR Clean-Up Kit or GenElute Gel Extraction Kit (Sigma-Aldrich). The DNA was quantified using a SimpliNano spectrophotometer (Biochrom, Harvard Bioscience, UK). Cloning procedures involved DNA digestion and ligation using FastDigest endonuclease restriction enzyme and T4 DNA ligase (1 U/µL) from Thermo Fisher Scientific, following the manufacturer’s instructions. Standard recombinant DNA manipulations were performed as described previously [28].

### Mutant generation and verification

Mutants were generated by electroporation transformation, which was conducted according to a previously described protocol [29]. In the gene silencing experiment, the wild-type R7B strain served as the recipient strain, and transformants were selected in the YNB medium. The control strain was established by transforming R7B with the vector pMAT1812, without any insert fragments. This vector utilizes a fragment from the carotenoid-producing gene *carB* as a reporter gene, as described previously [26].

For gene knockout experiments, disruption cassettes were introduced into the protoplasts of the MU402 strain (*pyrG^-^* and *leuA^-^*), initiating homologous recombination for gene replacement. The selection process took place on the MMC-selective medium through at least five vegetative cycles. This approach aims to enhance the proportion of transformed nuclei, considering that primary transformants are heterokaryons, owing to the presence of multiple nuclei in the protoplasts [26,30]. To confirm the genotypes of the transformants, a PCR-based strategy was employed using various primer combinations (Table S1). The PCR results provided insights into whether the marker gene *pyrG* had been integrated correctly into the target locus and whether the selected transformants were homokaryotic or heterokaryotic.

### Phenotypic analysis and microscopic imaging

To measure the vegetative growth of the strains, small pieces (~1 mm in diameter) of mycelia were inoculated onto the surfaces of medium plates. Vegetative growth was estimated by measuring the diameter of colonies every 24 h for 3 to 5 consecutive days [31]. The sporulation ability was measured following a previously described procedure using spores harvested from mature mycelia [32]. Sporulation ability is the ratio of the total number of spores divided by the surface area of colonies grown for 72 h at 26 °C under continuous light conditions.

To assess germination and polar growth, fresh vegetative spores of each strain were cultured in 25 ml of MMC liquid medium (pH 3.2) in a sterile Erlenmeyer flask, with the sporangial concentration adjusted to 10^6^ spores/mL. The cultures were grown at 26 °C with constant shaking (200 r.p.m.) for 7 h. Every hour, temporary specimens were prepared, observed and photographed using a computer-connected microscope system (ZEISS Axio Scope A1 with Axiocam 105 colour) and ZEN Microscopy software 2.6 (Carl Zeiss, Oberkochen, Germany). Five snapshots were taken for each sample to capture the microscopic field of view at the four corners and centre. These snapshots were used to extract data, including the total number of evaluated spores, the number of germinated spores and the length and width of the fungal hyphae. Spores with germ tubes longer than their diameter were considered germinated. To calculate the polarity index, ten germinating spores were measured in each snapshot using ImageJ software 1.54 g (National Institutes of Health, USA), resulting in a total of 50 germinating spores per sample [26].

### Virulence assays

Virulence assays in *Galleria mellonella* were performed as previously described, with minor modifications [26,33]. Briefly, sixth instar larvae were injected through the last pro-leg with 5000 vegetative spores or yeast-like cells in a volume of 10 µL of phosphate-buffered saline (PBS) and incubated at 30 °C in the dark. Untouched larvae and larvae injected with 10 µL of PBS served as negative controls. The positive control larvae were injected with spores of the wild-type R7B strain. Each group contained 10 larvae. Each experiment was repeated thrice. The survival of the infected larvae was monitored every 24 h for seven consecutive days.

### Statistical analysis

Data are expressed as mean ± standard deviation (SD). The data were stored, analysed and illustrated using Microsoft Excel and Origin 2021b. ANOVA analysis (α = 0.05) followed by the Tukey post-hoc test was applied to identify the statistically significant differences. To analyse the survival of wax moth larvae, a log-rank (Mantel–Cox) test was used to compare survival ratios between samples. In all tests, *P* values of less than 0.05 were considered statistically significant: *p<0.05, **p<0.01, ***p<0.001, ns *p*>0.05.

## Results

### Myo5B protein found in *M. lusitanicus* has the canonical structure of myosin V

To identify myosin V-encoding genes in *M. lusitanicus*, we performed BLASTp searches of the *M. lusitanicus* genome databases at the fungal genomics resource (MyCocosm, JGI) using the protein sequence of Mcmyo5 (ID 51513), which was identified previously [17]. Three myosin V protein homologs were identified and their encoding genes were randomly distributed in the genome of *M. lusitanicus*, including IDs 179665, 154 518 and 138 262.

**Fig. 1.**
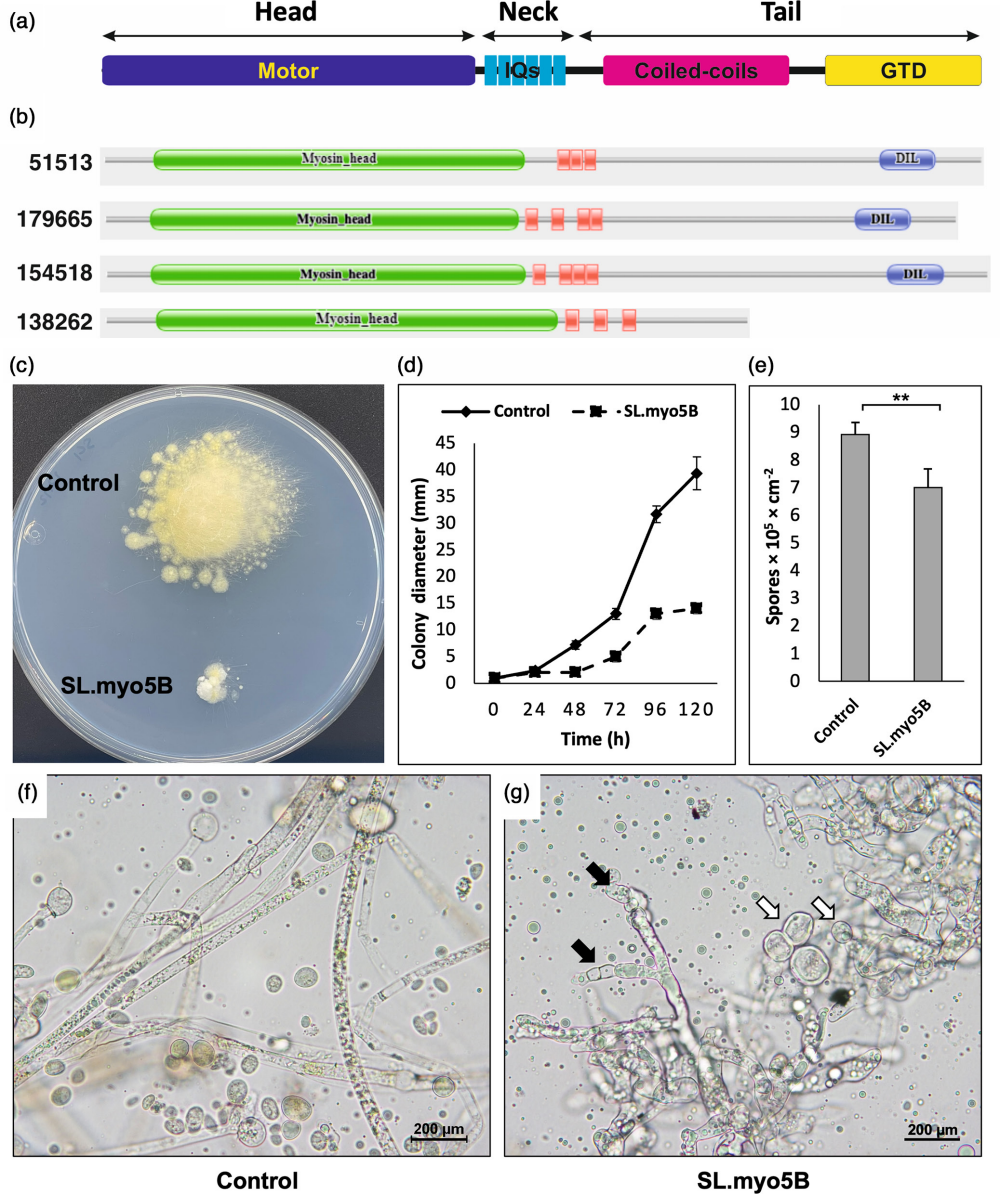
Functional analysis of the Myo5B protein in *M. lusitanicus* using RNAi. (**a**) General architecture of the myosin V protein, including the motor head domain, the neck and the tail region. (**b**) The domain structures of the protein IDs (51513, 179665, 154 518 and 138262) that were identified in the *M. lusitanicus* genome. (**c**) Colony of silencing phenotype (SL.myo5B) compared to the control (R7B.pMAT1812) strain at 120 h of incubation. Quantification of growth rate (**d**) and sporulation (**e**) of *myo5B*-silencing phenotypes compared to the control strain. (**f, g**) Microscopic images display morphological abnormalities of *myo5B*-silencing phenotypes compared to the control strain. Black arrows denote yeast-like cells; white arrows denote pseudo-septa in fungal hyphae. The colonies were grown on YNB medium (pH 2.8) at 26 °C under a continuous light condition. YNB, yeast nitrogenous base. **p<0.01.

Analyses of these three sequences using InterProScan revealed that all contained distinctive conserved domains of class V myosin heavy chains. They contained an N-terminal myosin motor head domain, several IQ calmodulin-binding motifs and a C-terminal tail domain with a coiled-coil morphology, except for ID 138262 (Fig. 1a, b). Hence, homologs with IDs 179665, 154 518 and 138 262 were designated *myo5B, myo5C* and *myo5D*, respectively (Fig. 1b). In this study, we focused on the function of the Myo5B protein, which has 1567 amino acids, with the locations of the domains and motifs mentioned in Table S3.

### Knockdown of the *myo5B* gene resulted in the reduction of growth rate and sporulation

RNAi was used to silence the target genes. A specific dsRNA expression vector (pMAT1812) was used to generate the specific silencing plasmid. To suppress the expression of the *myo5B* gene, RNAi plasmid pAT31 was generated by cloning a PCR fragment of the target gene into vector pMAT1812 [26]. After transformation, the obtained knockdown fungal strain showed yeast-like growth and a significant reduction in the growth rate (Fig. 1c, d). The difference in the growth rate between the two strains became clear after 48 h of growth on selective YNB medium. The sporangiophores generated by the *myo5B*-silencing phenotype were much fewer than those generated by the control strain (Fig. 1e) on a solid medium, indicating that the sporulation process of the knockdown strain was partially inhibited.

Notably, the *myo5B*-silencing phenotype showed abnormal hyphal morphology compared with that of the control strain. This silencing phenotype generated yeast-like cells with pseudosepta (Fig. 1f, g). The appearance of yeast-like cells suggests that inhibition of *myo5B* reduces the virulence of this fungus [18,27]. However, the silencing of the target gene was not stable. In particular, the knockdown of the *myo5B* gene generated different colonies with different growth rates and sporulation compared to the control strain. Therefore, it was necessary to generate a null mutant strain of the *myo5B* gene.

### Generation of *myo5B*∆ null mutant strain

**Fig. 2.**
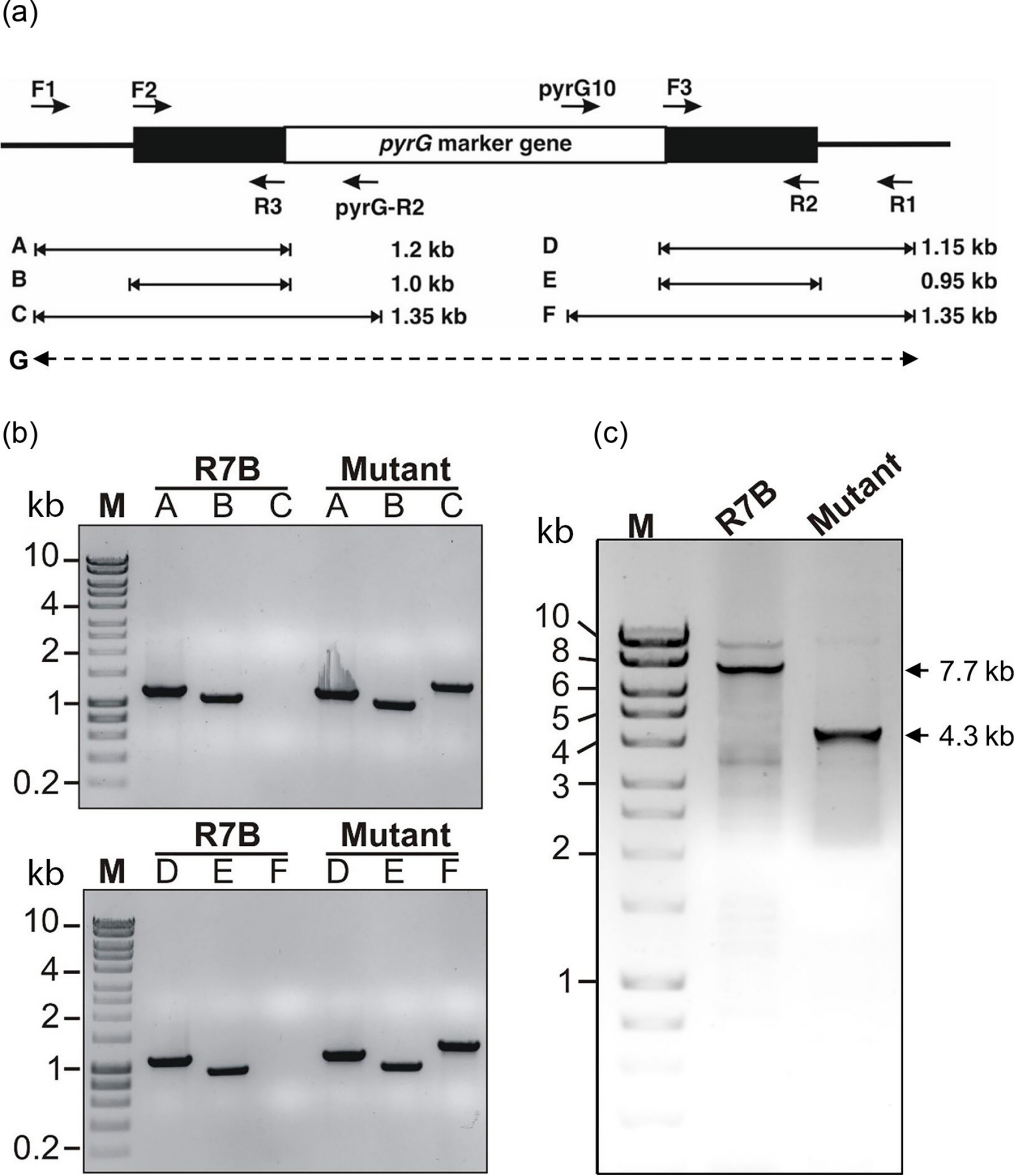
Verification of a knockout mutant using a PCR-based strategy. (**a**) Schematic diagram illustrating the location of primers at the target locus with expected product sizes in each PCR reaction. The black boxes denote the upstream and downstream segments of the target gene, and the white box denotes the marker gene pyrG in the disruption cassette. (**b**) PCR validation of transformants corresponding to *myo5B* compared to the wild-type R7B strain. The letters (A–F) indicate the PCR reactions with the primers mentioned in Fig. 2a. (**c**) Results of PCR (reaction G) were obtained to check the genotypes of the transformant. In these PCR reactions, the wild-type and *myo5B∆* strains generated bands of 7.7 and 4.3 kb, respectively.

A gene knockout investigation was conducted to delve deeper into the influence of the Myo5B protein in *M. lusitanicus*. To facilitate gene replacement through a double-crossover mechanism, disruption cassettes were generated by cloning the selective *pyrG* gene flanking the upstream and downstream fragments of the target gene. Six of the 15 original transformants were grown on selective MMC medium for five vegetative cycles, resulting in one knockout mutant strain (Fig. 2).

To confirm the accuracy of the integration sites and to determine the genotype of the mutants, a PCR-based technique was employed (Fig. 2a) with different combinations of DNA oligos at both ends of the target genes (Table S1). The results shown in Fig. 2b confirm that the target myo5B gene of the fungal mutant was correctly replaced by the selective marker, *pyrG*. Transformants from *myo5B* disruption exhibited DNA fragments corresponding only to the correct integration of the disruption fragment at the designated locus (Fig. 2b, c), indicating a homokaryotic status for the mutant allele. The *myo5B* gene was successfully eliminated from the *M. lusitanicus* genome. The validated knockout mutant strain was designated as Mc24. Nevertheless, this PCR-based approach cannot identify ectopic insertions that may arise alongside the integration of the replacement cassette. Theoretically, the likelihood of concurrent target gene replacement and ectopic insertion is low. In *Neurospora crassa*, the nuclei undergoing transformation via homologous recombination exhibit limited competence in ectopic integration [34]. Despite the low frequency, the generation of knockout mutants in this study yielded transformants harbouring multiple copies of the disruption cassette.

### Deletion of the *myo5B* gene reduces growth rate and vegetative sporulation

**Fig. 3.**
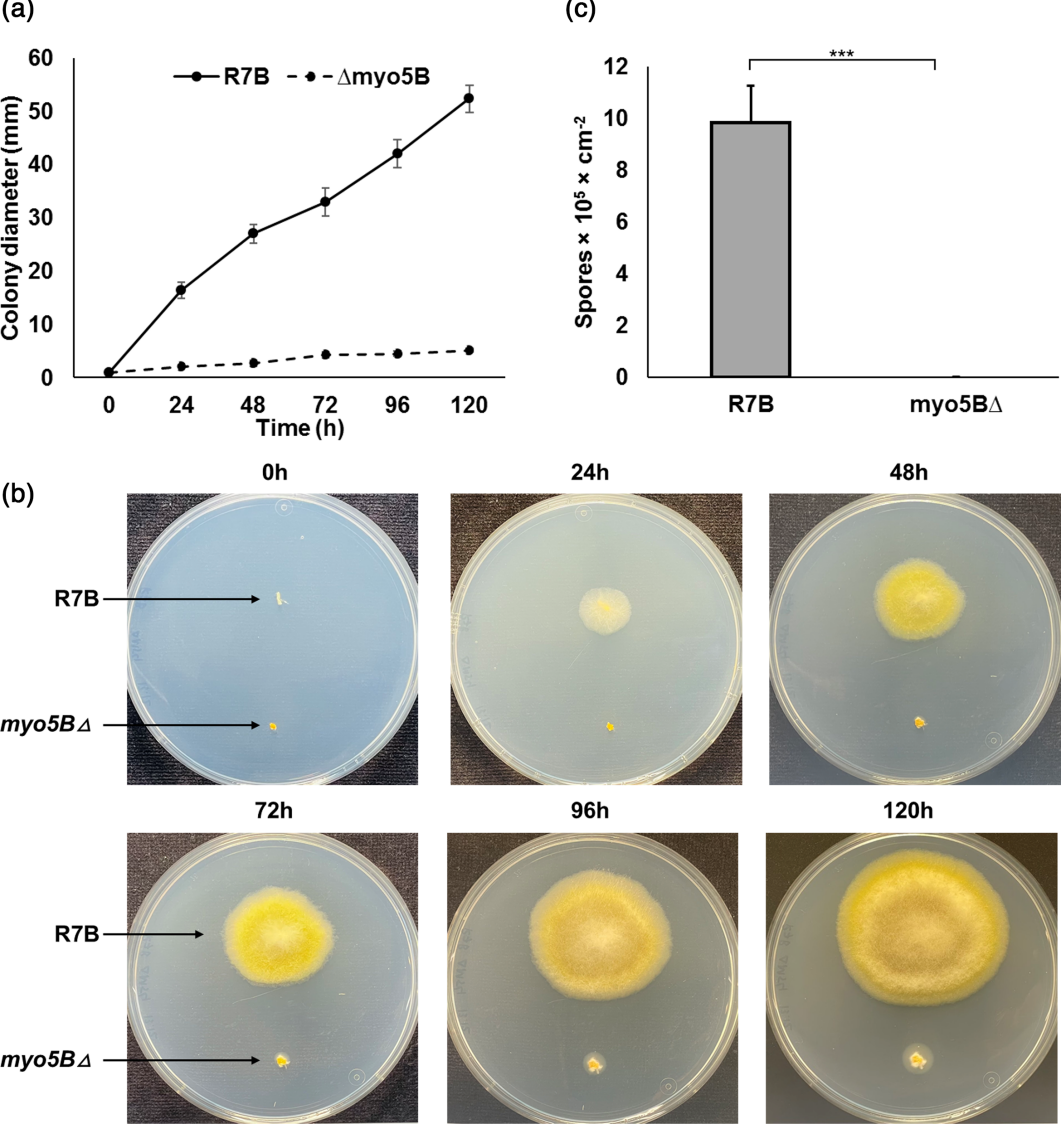
Reduction of growth rate and sporulation of the *myo5BΔ* mutant strain. (**a, b**) Growth rate of the wild-type and mutant strains during 120 h incubation on solid MMC medium, pH 3.2 and continuous light conditions. (**c**) Sporulation was quantified by calculating the total number of spores harvested from fungal colonies cultivated after 72 h growth on YPG solid medium with pH 4.5, under continuous light conditions at 26 °C. MMC, minimal media with casamino acids; YPG, yeast–peptone–glucose. ***p<0.001.

The results of the growth evaluation of mutant strains in solid MMC medium were highly consistent with the observations of silencing phenotypes (Figs 3 and 1). The diameters of fungal colonies were measured during 120 h to examine the growth rate of the *myo5BΔ* mutant compared to the wild-type R7B strain. The growth rate of the *myo5BΔ* mutant was strongly reduced compared with that of the wild-type strain on the MMC solid medium. At 120 h of culture, the colony diameter of the wild-type R7B was approximately 10 times that of the diameter of the *myo5BΔ* strain (Fig. 3a, b).

**Fig. 4.**
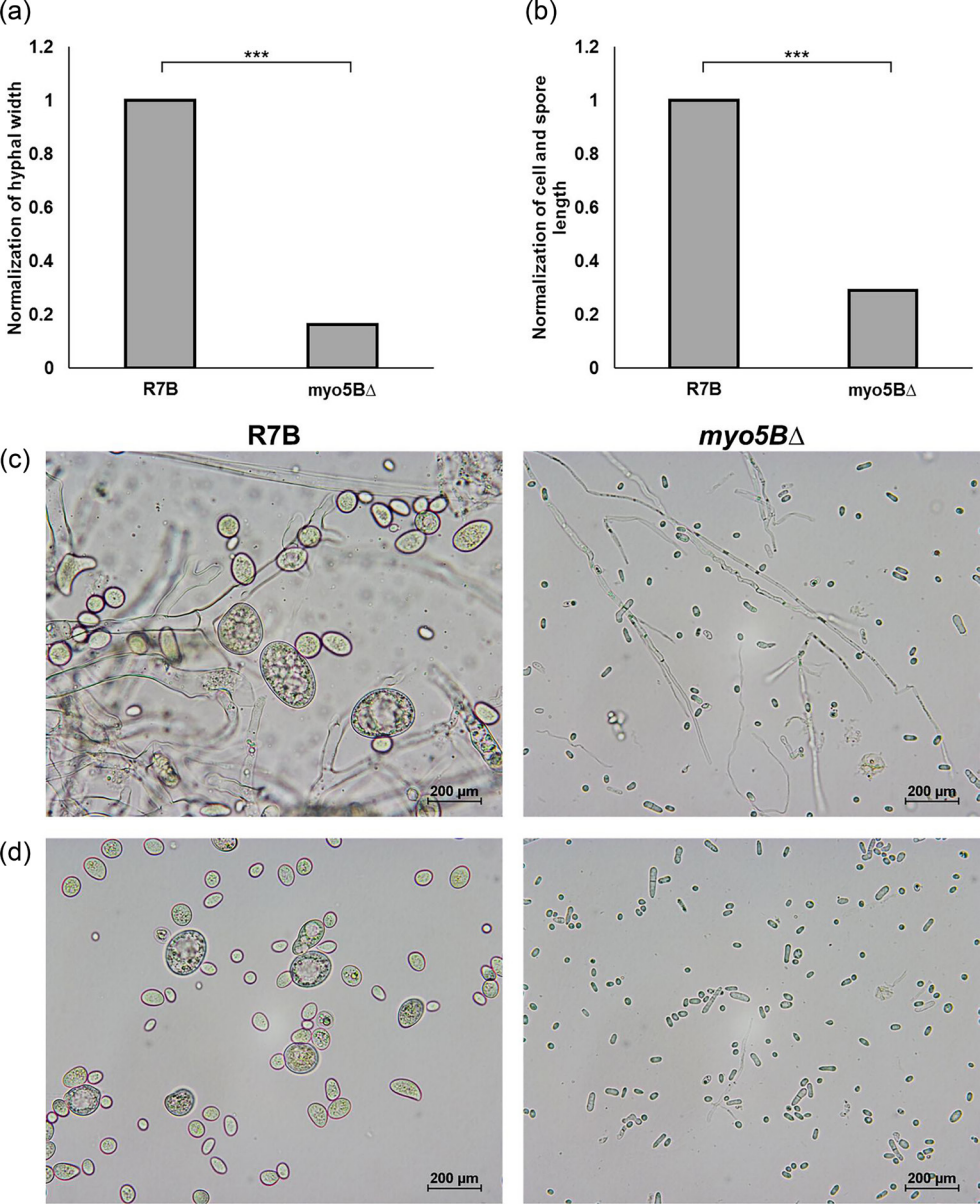
Reduction of hyphal width and spore/cell length of the *myo5BΔ* mutant strain. Normalization of the width of the filamentous hyphae (**a**) and the length of spores/cells (**b**) of the *myo5BΔ* mutant strain against the wild-type R7B strain. Morphology of mycelia (**c**) and spores and yeast-like cells (**d**) of the wild-type strain and the mutant strain, respectively. ***p<0.001.

Deletion of the *myo5B* gene most likely resulted in the loss of the ability to produce spores (Fig. 3c), since the mycelia of the mutant grown on MMC or rich YPG media only generated yeast-like cells but not spores. The mutant fungal colonies mostly contained yeast cells and small hyphae instead of the normal hyphal network of the wild-type strain. The yeast-like cells of the mutant were easily released from the mycelia when observed under a microscope (Fig. 4c, d). The mutant strain did not generate mature hyphae; therefore, it was unable to produce spores (Fig. 3c). Due to the lack of sporulation in the mutant, we were unable to perform a functional complementation experiment.

### Myo5B protein plays a key role in polarity growth and dimorphism switch of *M. lusitanicus*

**Fig. 5.**
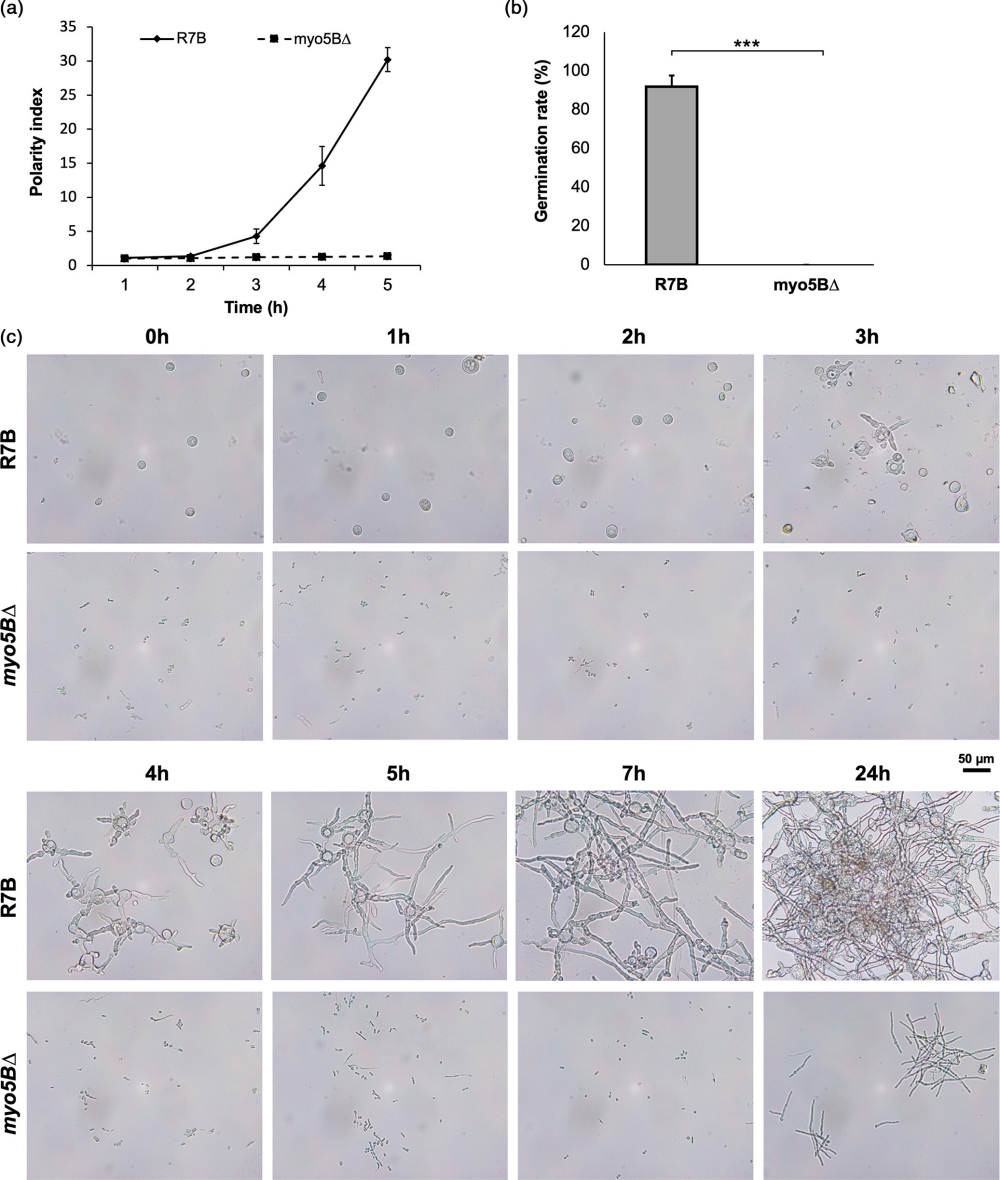
Deletion of the *myo5B* gene reduces the polarity index (**a**), germination and branching rate (**b, c**). The wild-type R7B and mutant strains *myo5B∆* were grown during 5, 7 and 24 h incubation in YPG liquid medium, pH 4.5, under conditions of 26 °C and agitation at 200 r.p.m. ***p<0.001.

The polarity indices of the wild-type and mutant strains were measured to evaluate the growth polarity of fungal cells. The polarity index is the quotient of cell length divided by cell width [35] and is an indicator of germination and hyphae formation. The polarity index can be used as an indicator of fungal growth and virulence [26,36]. In this study, the polarity index of the *myo5BΔ* mutant cells was considerably significantly reduced compared to the wild-type strain R7B, especially after 3 h of growth in liquid culture (Fig. 5a). The spores of the control strain started to germinate after 3 h of incubation and rapidly elongated the hyphae over the next several hours (the polarity index increased from ~5 to ~30). In contrast, the mutant cells did not germinate or produce any branches after 7 h of incubation, leading to considerably low polarization (Fig. 5). To determine whether the mutant eventually formed hyphae, the fungal strains were grown beyond 5 h, until 7 h and 24 h in liquid medium. The cells of the mutant strain could not form normal hyphae similar to the wild-type strain. After 24 h of growth in liquid YPG medium, the mutant cells had extended but still could not branch or produce regular hyphae (Fig. 5c).

The *myo5BΔ* mutant strain was not only reduced in polarity growth but also generated mycelia significantly smaller sizes (~1/6.3 times) than the wild-type strain (Fig. 4a and c). Mycelia of the mutant strain were smaller, shorter and less branched than those of the wild-type strain (Fig. 4c). Notably, the yeast-like cells obtained from the *myo5BΔ* mutant strain are strongly reduced in size (~1/2.5 times) compared to the spores of the wild-type strain (Fig. 4b and d). Dimorphism is a characteristic feature of pathogenic fungi [7]. During the growth process, the mutant *myo5BΔ* mostly likely produced only yeast-like cells instead of normal hyphal mycelia. This suggests that their virulence was also reduced [17]. Collectively, these findings suggest that Myo5B plays an important role in the development and morphology of *M. lusitanicus*.

### The *myo5BΔ* mutant lost its virulence

Virulence assessments of the fungal mutant strain *myo5BΔ* were conducted using *G. mellonella* larvae as a host, a widely used model for mucormycosis research [33]. Inoculation involved 5000 vegetative spores for the wild-type strain and yeast-like cells for the *myo5BΔ* mutant strain. The survival of infected larvae was observed at 24 h intervals for 7 consecutive days. Two negative control groups were monitored: non-injected individuals and larvae injected with PBS.

**Fig. 6.**
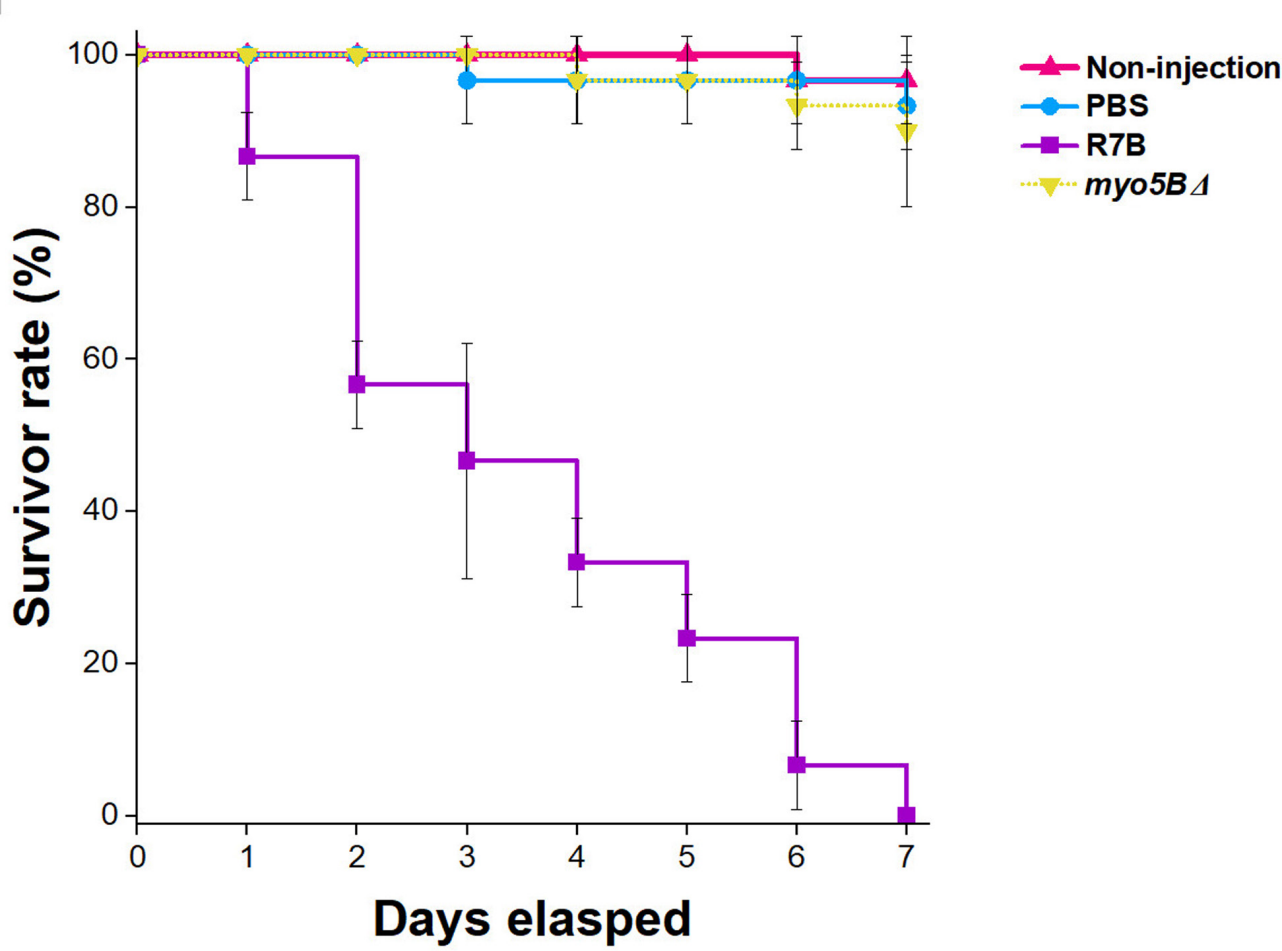
Results of virulence assays of the *myo5BΔ* mutant in the *G. mellonella* larvae. Injections contain 5000 vegetative spores or yeast cells per dose. The survivor rates of the hosts were measured 7 days after treatments.

The *myo5BΔ* mutant strain demonstrated complete avirulence, with the survival rate of infected larvae showing no significant difference from the negative control groups at all the time points monitored (*P* > 0.05, Fig. 6). In contrast, hosts infected with the wild-type R7B strain exhibited significantly lower vitality than those infected with the mutant strain at 2 days post-infection (*P* < 0.001, Fig. 6). This implies that the *myo5B* gene likely plays an important role in the pathogenesis of *M. lusitanicus*, contributing to delayed growth, reduced sporulation and abnormal hyphal morphology.

## Discussion

Myosin is a notable superfamily of molecular motors that use energy derived from ATP hydrolysis to produce forces for power movement on actin filaments in all eukaryotic cells [18,37]. Their members are grouped into several classes with diverse cellular functions [38]. Four classes of myosins with myosin motor domains have been found in fungi: myosin I, myosin II, myosin V and fungus-specific Chs [39]. Myosin motor proteins are more abundant in Mucoralean fungi than in other fungi. We previously identified 17 putative myosin homologs in the *M. lusitanicus* genome that displayed a significantly higher abundance of myosin motor molecules than other fungi [26]. These include four proteins classified as myosin V. The large number of myosin motor proteins in basal fungi implies their involvement in growth, development and other biological processes.

Class V myosins, one of the most extensively studied myosins, belong to a highly conserved protein family in eukaryotic species [40]. This class of myosins differs from others by having an extended neck and tail domain that allows dimerization. In humans, three genes encoding class V myosins have been identified among 38 myosin family genes [41]. In *Saccharomyces cerevisiae*, five myosin genes have been identified, including two class V myosin genes (*myo2* and *myo4*) [42]. In the dimorphic pathogenic fungus *U. maydis*, a single myosin class V protein encoded by *myo5* is involved in mating, hyphal growth and pathogenicity [22]. In this study, we found that *myo5B* (ID 179665) encodes a myosin class V protein in *M. lusitanicus*. The Myo5B protein has all the canonical conserved domains in the myosin class V family, including a myosin motor domain, four IQ motifs and a DIL domain. The absence of the Myo5 protein inhibits hyphal formation and results in yeast-like growth. This suggests that *Mucor* cells require this protein for normal hyphal growth.

For the initial analysis of the biological function of the target gene, the RNAi technique was applied. The *myo5B*-silencing phenotype showed a strong reduction in growth rate and sporulation compared to the control strain. For knockdown experiments, we used a dsRNA expression vector (pMAT1812), which was used in our previous study [26]. This vector contains a simple reporter gene fragment, *carB*, which generates the albino phenotype of *carB*-silencing strains. Therefore, the presence of white colonies and the alteration of the transformant phenotype indicated that the DNA insert was also expressed. However, this technique has the potential for off-target effects; therefore, it must be confirmed using deletion mutants of the target gene. However, the silencing phenotype was unstable. The unstable phenotypes of the *myo5B*-silencing strain could be explained by the presence of different numbers and expression levels of the RNAi plasmids inside the cells of transformants. In general, an increase in copy number and level of plasmid expression can increase the silencing effect [26]. This characteristic of the knockdown strain explains why the silencing phenotype still produces vegetative spores and why its growth performance was significantly different compared with the null mutant strain. In *M. lusitanicus*, the myosin class V family comprises several members with similar structures and domain architectures. However, they could play different functions in this fungus, as the *myo5B* gene could be completely deleted from its genome, similar to myosin V in other organisms, but not the previously identified *mcmyo5* (ID 51513) gene [17]. Both genes play important roles in hyphal morphology as well as the virulence of *M. lusitanicus*. These findings indicate that the biological functions of myosin V proteins are abundant, but not the same, in *Mucor* fungi. Myosin V proteins are involved in various cellular processes, including cargo translocation; therefore, they play a role in fungal polarity, growth and hyphal formation [20,22,24]. Further investigation of the biological functions of other members is required to better understand their functions in this fungus.

Dimorphism is a key characteristic of pathogenic fungi. The ability to switch between yeast and filamentous phases plays an important role in fungal virulence [7]. Filamentous fungi are defined by their ability to generate highly polarized hyphal growth [43]. In *M. lusitanicus*, the yeast-like form is less virulent than the hyphal form [8]. Similar to the disruption of Myo5 protein [17], the loss of Myo5B in *Mucor* resulted in the loss of cell polarity, as it generated a yeast-like morphology under normal growth conditions. However, this result is unlike that of *U. maydis*, in which the loss of Myo5 did not affect the tip growth of the hyphae and sporidia [22]. In contrast, deletion of the *myo5B* gene completely lost the capacity to produce asexual spores, which is similar to the disruption of the *mcmyo5* and *mcmyo2A* genes, members of myosin V and myosin II, respectively, in *M. lusitanicus* [17,26].

The absence of transporters responsible for delivering secretory vesicles to growth regions may account for the loss of polarized growth [44]. It is plausible that certain vesicles, identified as the specific cargo of Myo5 in *Mucor*, make up the apical vesicle crescent (AVC), a structure observed in the hyphae of *Mucorales*, serving as the organizing centre for hyphal growth and morphogenesis [45]. Numerous small vesicles are present in growing hyphal tips during spore germination and at sites of branch formation [46]. The location of the AVC in the hyphal tip aligns with the direction of hyphal growth. Therefore, the absence of the appropriate vesicle concentration at specific points during the germination of the *myo5B∆* mutant may hinder its polarized hyphal growth.

In addition, the *in vivo* virulence assay in the *G. mellonella* host system has shown a significant reduction of virulence in the *myo5B∆* mutant compared to that of the wild-type R7B strain. Seven days after infection, the insects gradually pupated, and most of the pupae survived. Therefore, the survival ratio of the insects did not change significantly after the experimental period. This result suggests that Myo5 plays an important role in *M. lusitanicus* pathogenesis, probably through its requirement for hyphal growth and sporulation, and confirms the relationship between yeast-like growth and hypovirulence [8,9]. Taken together, the Myo5B protein belonging to the myosin V family is likely to be a multitasking carrier involved in polarity growth, branching patterns, abnormal septation, sporulation and pathogenicity in the human pathogenic fungus, *M. lusitanicus*. Myo5B and other members of the myosin V family could be used as potential targets for future therapies to effectively treat mucormycosis.

## supplementary material

10.1099/mic.0.001482 Uncited Table S1.

## Abbreviations

AVC: apical vesicle crescent
Chs: chitin synthase
COVID-19: coronavirus disease 2019
LB: Luria Broth
MMC: minimal media with casamino acids
PBS: phosphate-buffered saline
RNAi: RNA interference
SD: standard deviation
YNB: yeast nitrogenous base
YPG: yeast–peptone–glucose
