# Supplementary material for: Myo5B plays a significant role in the hyphal growth and virulence of the human pathogenic fungus Mucor lusitanicus
Source: Microbiology (Reading). 2024 Jul 29;170(7):001482. doi: 10.1099/mic.0.001482 (PMC11286281; doi:10.1099/mic.0.001482)
Supplement: Uncited Table S1. [file mic-170-01482-s001.pdf]

Supplementary data

Table S1. List of primers used in this research

**Primers**	**Sequence (5´- 3´)**	**Use**
PU	GTTGTAAAACGACGGCCAGT	Colony PCR
peuka-1	CATGAAGTGTGAGACATTGCG	Colony PCR
F-pyrG	TGCCTCAGCATTGGTACTTG	Selectable marker amplification
R-pyrG	GTACACTGGCCATGCTATCG	Selectable marker amplification
pyrG-R2	ATCCCACCAGAAGGAGTACATGG	Mutant comprobation
pyrG10	GGCAGAAGGGAGGAGGCACACGC	Mutant comprobation
Fsl-1	TT**CTCGAG**CGGCTTCGTACTGAACATCCAG	*myo5B* silencing
Rsl-1	TT**GCGGCCGC**TACACTCAGGGCACCAAGGCTT	*myo5B* silencing
F1	AGTTGCCGCAGACAGGAGCA	*myo5B* disruption
F2	TT**CTGCAG**CACAGACCAAGACAATGTGGGGTA	*myo5B* disruption
F3	*CGATAGCATGGCCAGTGTAC*CTCTTTCCGGTTTGTTGTGCCAAA	*myo5B* disruption
R1	CAGCACCATCAGAAGCGCCTT	*myo5B* disruption
R2	TT**CTGCAG**TGCCATCCATGCCCATCGTG	*myo5B* disruption
R3	*CAAGTACCAATGCTGAGGCA*CATCTATGACGAGTATCCAAGCAC	*myo5B* disruption

The recognition sites of restriction enzymes were indicated by bold regions. The overlapping fragments with *pyrG* selective marker gene were indicated by italic and underlined regions.

Table S2. Strains and plasmids used in this research

**Strain**	**Genotype**	**Phenotype/ Description**	**Source**
R7B	*leuA*$^{-}$	Leu-, virulent; used as the wild-type strain	University of Murcia, Spain
MU402	*leuA*$^{-}$, *pyrG*$^{-}$	Leu-, Ura-, virulent; used as the receipient strain	University of Murcia, Spain
Control	*leuA*$^{-}$, pMAT1812(*leuA*$^{+}$)	Leu+, R7B strain transformed by pMAT1812	This study
R7BpAT31	*leuA*$^{-}$, pAT31(*leuA*$^{+}$)::*myo5B*	Silencing phenotype of *myo5B* gene	This study
Δ*myo5B*	*leuA*$^{-}$, *myo5B*::*pyrG*	Knock-out mutant strain of *myo5B* gene	This study

**Plamids**	**Description**	**Source**
pMAT1812	Approximately 9.5kb in size, contains two opposite promoters, selective marker: *amp*^{R}, *leuA,* reporter gene *carB*	University of Murcia, Spain
pJET1.2/Blunt	Approximately 3kb in size, selective marker: *amp*^{R}, used for cloning experiments	Thermo Scientific
pAT31	The RNAi plasmid used to knock-down *myo5B* gene	This study
pAT47	Containing disruption cassette, which is a marker pyrG gene flanking by upstream and downstream regions of the *myo5B* gene (~1kb each)	

Table S3. The locations of the domains and motifs found in Myo5B protein

Family	Description	Entry type	Clan	Envelope		Alignment		E-value
				Start	End	Start	End	
**Myosin_head**	Myosin head (motor domain)	Domain	CL0023	82	761	82	761	4.2e-266
**IQ**	IQ calmodulin-binding motif	Motif	CL0220	777	797	778	796	0.14
**IQ**	IQ calmodulin-binding motif	Motif	CL0220	825	845	825	844	2.2e-06
**IQ**	IQ calmodulin-binding motif	Motif	CL0220	873	893	873	893	0.00042
**IQ**	IQ calmodulin-binding motif	Motif	CL0220	896	915	898	914	0.11
**DIL**	DIL domain	Family	n/a	1384	1485	1384	1483	1.3e-27
